# Braiding traditional ecological knowledge and Western science in the management of freshwater social-ecological systems: a systematic map protocol

**DOI:** 10.1186/s13750-025-00371-8

**Published:** 2025-10-14

**Authors:** Ronald J. Maliao, Béla Tóthmérész

**Affiliations:** 1 Department of Ecology, Pál Juhász-Nagy Doctoral School of Biology and Environmental Sciences, University of Debrecen, Debrecen, 4032 Hungary; 2 Aklan State University, New Washington, Aklan, 5016 Philippines

**Keywords:** Aquatic biodiversity, Co-production, Co-management, Conservation planning, Evidence synthesis, Fisheries, Indigenous and local knowledge, Knowledge integration, Local knowledge, Natural resource management

## Abstract

**Background:**

Freshwater ecosystems are globally imperiled, with monitored vertebrate populations showing an average 83% decline since 1970. Braiding Traditional Ecological Knowledge (TEK) with Western science is increasingly recognized by global bodies like the IPBES (Intergovernmental Science-Policy Platform on Biodiversity and Ecosystem Services) as essential for achieving the transformative change needed to address this crisis. This systematic map provides a comprehensive, global synthesis of the diverse methodologies used for this purpose by answering the primary question: What is the evidence base for methodologies (approaches, frameworks, or models) that braid the TEK of Indigenous and local communities with Western science in the planning, management, monitoring, or assessment of freshwater social-ecological systems? The resulting synthesis is intended to empower researchers, practitioners, and policymakers to design more effective and equitable management strategies.

**Methods:**

Following Collaboration for Environmental Evidence (CEE) guidelines, our protocol employs a multi-layered search strategy across three core bibliographic databases, targeted grey literature sources (including dissertations and key organizational websites), and a supplementary review-centric snowballing search. Records will be screened for eligibility in a two-stage process (Title/Abstract and Full-text) with robust consistency checking to ensure transparency and minimize bias. Data from included articles will be coded using a detailed protocol designed to answer our secondary questions and build a typology of knowledge braiding methodologies. The systematic map’s outputs will include a narrative synthesis identifying knowledge gaps and clusters, a comprehensive public database of included studies, and a suite of interactive data visualizations.

**Supplementary Information:**

The online version contains supplementary material available at 10.1186/s13750-025-00371-8.

## Background

Global freshwater ecosystems are critical hubs of biodiversity and provide vital ecosystem services, yet they are among the most imperiled habitats on Earth [1–3]. This vulnerability is starkly evident in recent biodiversity declines. Monitored freshwater vertebrate populations have plummeted by an average of 83% since 1970 [4], and the IUCN Red List indicates that one in four assessed freshwater species now faces extinction [5]. These severe declines are driven by key anthropogenic pressures, including habitat degradation, pollution, and climate change [1, 6]. Addressing this crisis requires robust conservation strategies to prevent further biodiversity loss, and safeguard essential ecosystem services [2, 6].

Effective management of these freshwater social-ecological systems, however, is often constrained by significant knowledge gaps. A primary constraint is the absence of historical baselines for assessing current trends, a problem worsened by the historical neglect of freshwater research [3, 7–9]. Furthermore, conventional scientific monitoring is often limited in space and time, failing to capture key long-term or fine-scale ecological dynamics [8, 10, 11]. These data challenges are compounded by the Shifting Baseline Syndrome (SBS) [12], where each new generation normalizes a more degraded state, obscuring long-term trends [3, 13]. Together, these limitations impede the design of effective conservation interventions and highlight an urgent need to braid diverse knowledge systems.

We adopt the term “braiding”, following the conceptual work of scholars such as Kimmerer [14], to describe bringing together two distinct but complementary knowledge systems: Traditional Ecological Knowledge (TEK) and Western science. Unlike the term “integration” which can imply the assimilation of one system into another, the metaphor of braiding suggests that both TEK and Western science retain their distinct integrity. Like the individual strands of a braid, they combine to create a stronger, more robust understanding for management action.

For this systematic map, we define TEK as the cumulative, place-based body of knowledge, practices, and beliefs about the environment [15–20]. It is held by Indigenous Peoples (IPs), defined by the UN as descendants of a region’s pre-colonial inhabitants, and by local communities who hold a deep, multi-generational connection to a specific place [21]. This body of knowledge is a key component of what bodies like IPBES refer to more broadly as Indigenous and Local Knowledge (ILK) [15, 16]. We contrast TEK with Western science, which we define as the formal, hypothesis-driven methodologies used to assess and manage social-ecological systems.

Braiding TEK with Western science creates a powerful synergy for managing natural resources, particularly in data-scarce regions [8, 18, 22]. By design, such a pluralistic approach allows TEK to address the common gaps in Western science, offering crucial insights into ecological history, population trends, and sustainable practices that are otherwise often missed [11, 22]. Crucially, effective and ethical engagement requires recognizing Indigenous Peoples and Local Communities (IPLCs) as essential partners and co-producers of knowledge, not merely as informants [14–19, 23]. This commitment to knowledge braiding is now a recognized priority in global policy frameworks, including assessments by IPBES [15].

The foundational mandate for knowledge braiding was established at the landmark 1992 Rio Earth Summit, where Agenda 21 called for recognizing and strengthening the role of Indigenous Peoples and their knowledge (Chap. 26) [24]. This political commitment was legally codified in the Convention on Biological Diversity’s Article 8(j), which obligates parties to respect and preserve traditional knowledge [25]. This mandate is cemented in the Kunming-Montreal Global Biodiversity Framework, whose Target 22 requires the full and effective participation of IPLCs and the integration of their knowledge. The commitment is reiterated most recently by the 2024 IPBES Thematic Assessment, which calls for the “weaving of Indigenous and local knowledge in decision-making” [15].

To operationalize these international mandates, policymakers and practitioners require a clear synthesis of existing braiding methodologies. While valuable reviews on TEK exist [26–28], a comprehensive synthesis of the methodologies for braiding TEK with Western science in the freshwater management nexus constitutes a critical gap. This systematic map [29] is designed to provide this foundational evidence base by charting the different approaches, frameworks, and models that have been employed or proposed globally. Ultimately, this evidence base will empower researchers, practitioners, and policymakers to design more effective and equitable management strategies for freshwater social-ecological systems. For this review, we define these systems broadly to include all inland aquatic environments, from freshwater rivers and lakes to transitional brackish habitats.

## Objective of the review

The objective of this review is to systematically map the evidence base for methodologies that braid Traditional Ecological Knowledge (TEK) with Western science in the planning, management, monitoring, or assessment of freshwater social-ecological systems. By identifying, collating, and describing these methodologies, our analysis will chart the research landscape to identify key knowledge gaps (such as under-represented geographic regions) and knowledge clusters (like commonly applied frameworks). For this review, “methodologies” refers to the full spectrum of braiding strategies, including the guiding Approaches, conceptual Frameworks, and specific Models as detailed in the ‘Concept’ component of our primary question.

### Primary question

What is the evidence base for methodologies (approaches, frameworks, or models) that braid Traditional Ecological Knowledge (TEK) of Indigenous and local communities with Western science in the planning, management, monitoring, or assessment of freshwater social-ecological systems?

### Primary question components

Our primary question is structured using the Population, Concept, and Context (PCC) framework [30], which defines the key components of our review:

1. Population: Studies focusing on the Traditional Ecological Knowledge (TEK) held by Indigenous Peoples, local communities, or specific resource-dependent groups, such as artisanal fishers.
2. Concept: Methodologies for braiding TEK with Western science. These include guiding approaches (the philosophical stances for collaboration, such as co-production), conceptual frameworks (the structured processes that guide braiding, such as Two-Eyed Seeing), or specific models (the tangible tools or outputs, such as participatory maps). The application of these methodologies must be within freshwater planning, management, monitoring, or assessment.
3. Context: Inland aquatic social-ecological systems globally. This scope includes freshwater environments and transitional brackish habitats such as estuaries and deltas.

### Secondary questions

Our analysis will extract data to characterize the evidence base and build a typology of knowledge braiding methodologies. The following seven set of questions will guide our analysis:

1. Bibliographic and geographic characteristics: What are the publication trends over time, the geographic locations of the studies, and the affiliations of the lead authors?
2. Ecological context: What freshwater ecosystem types and what taxonomic groups are the focus of the research?
3. Knowledge-holding communities: Are the communities identified as Indigenous Peoples, non-Indigenous local communities, or specific resource-user groups?
4. Type of TEK braided: Is the TEK braided primarily practical/empirical (such as species knowledge), institutional (such as customary rules), or symbolic/spiritual in nature?
5. Purpose and stage of braiding: What is the stated purpose of the knowledge braiding and at what stage of the process does it occur?
6. Tools, techniques, and ethics: What research designs, specific tools, and ethical protocols are reported?
7. Directionality, barriers, and enablers: What is the directionality of the braiding process (such as TEK informing science) and what are the key reported barriers and enablers to this process?

## Methods

This systematic map protocol adheres to the guidelines of the Collaboration for Environmental Evidence (CEE) [31] and is reported in accordance with the Reporting Standards for Systematic Evidence Syntheses (ROSES; Additional file 1) [32].

### Searching for articles

Our search strategy employs a multi-layered approach designed to be comprehensive, transparent, and replicable. The strategy consists of three complementary layers: a core search of bibliographic databases, a targeted search for grey literature, and a supplementary snowballing search.

### Comprehensiveness of search

We constructed our search syntax using standard Boolean logic based on the four conceptual blocks of our primary question. Within each block, we combined synonyms with the ‘OR’ operator and enclosed multi-word phrases in quotation marks. We then joined the four conceptual blocks using the ‘AND’ operator to ensure that each retrieved record contained at least one term from each block. We also employed proximity operators to refine the search where appropriate.

To validate our syntax, we developed and iteratively tested five versions in Scopus against a pre-defined test list of 20 key articles (Additional File 2). The initial, more specific searches (Searches 1–3) proved insufficiently sensitive, retrieving only 4, 8, and 13 of the test articles, respectively Search 4 was the first to achieve 100% sensitivity, retrieving all 20 articles with a total of 1417 results. Although the final and most sensitive search (Search 5) also retrieved all 20 articles, it returned a higher number of hits (1687). Therefore, following our a priori rule to select the most efficient syntax that achieved full sensitivity, we selected Search 4 as our final, optimal search string (see Additional file 3 for full details). This choice ensures a validated balance between comprehensiveness and a feasible workload for screening.

### Search terms and languages

Our final search string, presented in Scopus syntax, is provided in Table 1. The full, unabridged strings for all databases and the details of their development are provided in Additional file 4. A search update will be conducted if more than two years pass between the initial search (10 September 2025) and the final manuscript submission.

The search will be limited to articles published in English, a decision based on the linguistic proficiency of the review team. We consider this necessary because the nuanced, culturally-embedded terminology of TEK research requires a high level of fluency to avoid misinterpretation and ensure data accuracy. We acknowledge this decision may introduce a geographic bias, a limitation that will be addressed in the discussion of the final systematic map.

**Table 1:** Search strategy components

Conceptual block	Scopus search syntax
Population	TITLE-ABS-KEY( ((“local ecological knowledge” OR “traditional ecological knowledge” OR TEK OR “traditional environmental knowledge” OR “aborigin* knowledge” OR “ancestral knowledge” OR “artisanal knowledge” OR “customary knowledge” OR “ethnoecolog*” OR “ethnoichthyolog*” OR “ethnoscien*” OR “ethnotaxonom*” OR “ethnozoolog*” OR “fisher* ecological knowledge” OR “fisher* knowledge” OR “folk knowledge” OR “generational knowledge” OR “hunter* knowledge” OR “indigenous ecological knowledge” OR “indigenous traditional knowledge” OR “indigenous science” OR “indigenous knowledge” OR IK OR “lay knowledge” OR “native knowledge” OR “native science*” OR “place-based knowledge” OR “tribal knowledge” OR “communit* knowledge” OR “ethnobiolog*” OR “farmer* knowledge” OR “fishing communit*” OR “indigenous communit*” OR “indigenous people*” OR “local communit*” OR “local wisdom” OR “native communit*” OR “traditional communit*” OR “traditional knowledge”)) AND
Concept: Braiding	((“braid*” OR “co-management” OR “participatory mapping” OR “adaptive co-management” OR “co-production of knowledge” OR “complementary knowledge” OR “knowledge co-production” OR “knowledge mobilization” OR “knowledge pluralism” OR “knowledge socialization” OR “multiple evidence base” OR “participatory assessment” OR “participatory monitoring” OR “transdisciplinary science” OR “two-eyed seeing” OR “citizen science” OR “participatory approach” OR “participatory governance” OR “stakeholder engagement” OR “transdisciplinary research” OR (community-based W/10 monitor*) OR (community-based W/10 manag*) OR (community-based W/10 assess*) OR (entwin* W/10 knowledge) OR (pluralis* W/10 knowledge) OR (weav* W/10 knowledge) OR (bridge* W/10 knowledge) OR (collaborat* W/10 indigenous) OR (collaborat* W/10 local) OR (collaborat* W/10 native) OR (collaborat* W/10 communit*) OR (collaborat* W/10 tribal) OR (collaborat* W/10 fisher*))) AND
Concept: Application	((“protected area” OR “fish* manag*” OR “environmental impact*” OR “environmental assessment*” OR “adaptive manag*” OR “biodiversity” OR “climate change” OR “climate change adapt*” OR “conservation of natural resources” OR “ecolog*” OR “ecosystem resilience” OR “ecosystem*” OR “environmental change*” OR “environmental protection” OR “fish*” OR “manag* practice*” OR “nature conserv*” OR “resource manag*” OR “social-ecological system*” OR “water quality” OR “water resource* planning” OR “access and benefits sharing” OR “biocultural diversity” OR “customary manag*” OR “socio-institutional transform*”)) AND
Context	((“spring*” OR “stream*” OR “river*” OR “delta*” OR “pool*” OR “reservoir*” OR “lake*” OR “fen*” OR “aquatic” OR “freshwater” OR “watershed*” OR “catchment*” OR “wetland*” OR “estuar*” OR “pond*” OR “marsh*” OR “bog*” OR “creek*” OR “lacustrine” OR “floodplain*” OR “riparian” OR “inland water*” OR “swamp*” OR “limnolog*” OR “peatland*” OR “headwater*” OR “lotic” OR “lentic” OR “phytotelmata”)) )

The full search query is constructed by combining the four conceptual blocks with the ‘AND’ operator. The syntax shown is for Scopus and was adapted for other databases using specific field codes, such as TS = for Web of Science and NOFT for ProQuest

### Publication databases to be searched

The search for peer-reviewed studies will be conducted across three core bibliographic databases: Web of Science (WoS) Core Collection, Scopus, and the ProQuest Natural Science Collection. We selected these databases to provide comprehensive coverage, leveraging WoS for its curated indexing, Scopus for its extensive international coverage, and ProQuest for its specialized environmental focus. All searches will be performed using the subscription of the University of Debrecen. Our subscription to the WoS Core Collection includes 10 indices, most notably the Science Citation Index Expanded (SCI-EXPANDED) and the Social Sciences Citation Index (SSCI). Our search of the ProQuest Natural Science Collection covers the Agricultural Science, Biological Science, Earth, Atmospheric & Aquatic Science, and Environmental Science databases. Based on our scoping tests, these three databases yield 3,464 articles. The full list of all included indices and databases is provided in Additional file 4, along with the database-specific search strings.

### Search engine

As a supplementary search to enhance comprehensiveness, we will use Google Scholar (GS). Using Publish or Perish (PoP) [33], we will screen the top 1,000 relevance-sorted results from a simplified, title-only search designed to increase specificity [34]. The full search string used for this process is provided in Additional file 4. Our scoping test of this search yielded 403 potentially relevant articles.

### Grey literature searches

Our search for grey literature will employ a tiered strategy to balance comprehensiveness with feasibility. The primary tier is a systematic search targeting doctoral dissertations in the ProQuest Dissertations & Theses Global database. This focus on dissertations ensures methodological consistency, as they follow a standardized academic structure suitable for our data extraction protocol. The second tier is a targeted search of key international organizational websites (see next section) to identify relevant reports and publications from the policy and practitioner spheres.

### Organizational websites

To supplement our search of the academic literature, we will conduct a targeted grey literature search of key organizational websites. This search is designed to capture important policy documents and strategic frameworks not found in peer-reviewed journals. On each website listed in Table 2, we will perform a keyword search using our core terms and screen the first 100 relevance-sorted results for eligibility.

**Table 2:** Organizations targeted for the grey literature search

Intergovernmental bodies
• United Nations Educational, Scientific and Cultural Organization (UNESCO) Local and Indigenous Knowledge Systems (LINKS) [35] and Intangible Cultural Heritage (ICH) [36] • Intergovernmental Science-Policy Platform on Biodiversity and Ecosystem Services (IPBES) [37] • Convention on Biological Diversity (CBD) [38] • United Nations Framework Convention on Climate Change (UNFCCC) Local Communities and Indigenous Peoples Platform (LCIPP) [39] • Food and Agriculture Organization (FAO), specifically the publications of the Fisheries and Aquaculture Division [40] • Ramsar Convention on Wetlands Secretariat [41] • International Union for Conservation of Nature (IUCN) [42]
NGOs and research centers
• World Wide Fund for Nature (WWF) [43] • The Nature Conservancy (TNC) [44] • WorldFish [45]

### Targeted searches

To further enhance comprehensiveness, we will employ a strategic, ‘review-centric’ snowballing approach. This method uses the set of systematic and scoping reviews identified as highly relevant to our eligibility criteria during the full-text screening phase. This two-pronged strategy consists of backward snowballing, where we will manually check the reference lists of these key reviews, and forward snowballing, where we will use Google Scholar to screen newer articles that have cited them. We justify this efficiency-focused choice on the principle that such reviews serve as high-yield ‘integrative nodes’ of curated literature, allowing for a feasible yet comprehensive analysis of the most relevant study clusters. All articles retrieved through this process will undergo our standard de-duplication and screening procedures.

## Article screening and study eligibility criteria

### Screening process

Following the search, all retrieved records will be collated in EndNote (v.21.5) for metadata management and deduplication. The final, deduplicated set of records will then be imported into the systematic review software Colandr [46] for a two-stage screening process:

1. Title and Abstract (T/A) screening: Articles will first be screened at the title and abstract level against the established eligibility criteria. If there is any uncertainty regarding an article’s relevance, it will be conservatively retained for the next stage.
2. Full-text screening: All potentially relevant articles will be retrieved as full texts and screened again using the same criteria. A record of all articles excluded at this stage will be kept, along with the specific reasons for their exclusion, in line with ROSES reporting standards.

### Consistency checking

Prior to the main screening at each stage (T/A and Full-text), we will conduct a two-part consistency check. First, we will perform a calibration exercise. Two reviewers will independently screen a pilot subset of at least 50 articles. We will quantify inter-reviewer agreement using Cohen’s Kappa, and a score below 0.6 will trigger a discussion to refine the eligibility criteria for clarity [47]. This calibration will be repeated with new subsets until the agreement threshold is met.

Second, once consistency is established, a single reviewer will screen the remaining articles. To ensure ongoing reliability, a second reviewer will independently screen a random subset of at least 20% of the articles at both the T/A and Full-text stages [29, 34]. All disagreements during this process will be resolved through discussion to reach a consensus [26]. Reviewers who are authors of an article under consideration will declare a conflict of interest and will not participate in any inclusion decisions for that article.

### Eligibility criteria

To be included in the systematic map, an article should meet the following inclusion criteria, which are structured according to the components of the primary question.

1. Eligible population

- Inclusion criteria: The study should focus on Traditional Ecological Knowledge (TEK) or its widely recognized synonyms, such as Indigenous Knowledge and Local Knowledge. The evidence should pertain to the knowledge systems as they are held, articulated, or practiced by Indigenous peoples, local communities, or other resource-dependent groups, such as artisanal and recreational fishers.
- Exclusion criteria: A study will be excluded if it: (1) is a purely biophysical or ecological study that does not include a TEK; or (2) focuses on knowledge systems of industry or government experts without a corresponding local or Indigenous knowledge component.

2. Eligible concept

- Inclusion criteria: The study must describe, apply, propose, or assess a methodology that involves the combination of TEK and Western science for planning, management, monitoring, or assessment. This includes studies that explicitly label the process with terms like “braiding” or “integration,” as well as those that demonstrate the process without using specific terminology. The full spectrum of these braiding strategies is of interest, from guiding principles and conceptual frameworks (such as Two-Eyed Seeing) to specific tools and models (like participatory mapping).
- Exclusion criteria: A study will be excluded if: (1) TEK is mentioned only peripherally (i.e., it is mentioned without being substantively integrated into the study’s objectives, methods, results, or interpretation); or (2) it describes a methodology that is applied to a domain outside of freshwater environmental planning, management, monitoring, or assessment.

3. Eligible context

- Inclusion criteria: The study must be situated within an inland aquatic social-ecological system anywhere in the world. Eligible contexts include both freshwater environments (such as rivers, lakes, and wetlands) and transitional brackish habitats (like estuaries, deltas, and saltmarshes).
- Exclusion criteria: A study will be excluded if its context is purely marine or purely terrestrial, without a clear and direct link to a freshwater or estuarine environment.

4. Eligible study types

- Inclusion criteria: The study must present primary data or analysis derived from peer-reviewed scientific articles and specific forms of grey literature, including doctoral dissertations and reports from our predefined list of international bodies. There are no restrictions on publication date, but all included articles must be published in English.
- Exclusion criteria: Editorials, opinion pieces, letters, books, and book reviews will be excluded. Secondary literature (review articles, systematic maps) will also be excluded from the final data extraction; however, any review article that meets our eligibility criteria will be set aside and used as a source for our supplementary snowballing search.

### Study validity assessment

In line with CEE guidelines for systematic maps [26, 27], we will not conduct a formal critical appraisal of individual articles. Our primary objective is to map the breadth of the evidence base, not to determine the robustness of individual findings. However, to provide transparency and a foundation for future reviews, we will extract and code key methodological details from each study. This will include information on study design (such as qualitative or quantitative) and data collection methods (like interviews or ecological sampling), which will be presented in the final map to allow readers to understand the methodological landscape of the field.

### Data coding strategy

We will extract data from all included articles using a detailed coding protocol and a pre-designed data sheet (see Additional File 5). All coded data will be taken directly from the published text; we will not contact authors for missing information. To answer our secondary questions, we will code the following key categories for each study: (1) Bibliographic and geographic characteristics, (2) Ecological context, (3) Knowledge-holding communities, (4) Type of TEK braided, (5) Purpose and stage of braiding, (6) Tools, techniques, and ethics, and (7) Directionality, barriers, and enablers.

Prior to the main data extraction, we will conduct a calibration exercise to ensure the consistent application of our coding protocol. Two reviewers will independently code a pilot subset of at least 50 of the included articles. Following this, the reviewers will meet to compare their coding, discuss every discrepancy, and refine the definitions in the coding protocol until a consensus is reached. This process ensures a shared and rigorous understanding of all coding variables before the remaining articles are coded by a single reviewer.

### Study mapping and presentation

We will present our findings through three primary outputs: a narrative synthesis, a public database, and a suite of interactive data visualizations. The narrative synthesis will address our secondary questions to build an evidence-based typology of braiding methodologies. To do this, we will identify knowledge gaps and clusters using a multi-method approach that combines descriptive statistics, cross-tabulated heatmaps, and keyword co-occurrence network analysis [48]. These analyses will be presented through interactive data visualizations, including evidence atlases created with EviAtlas [49], and will be supported by a comprehensive public database containing all coded metadata to ensure transparency. Together, these outputs will provide a consolidated evidence base to advance the effective and equitable braiding of TEK in freshwater management, and the final report will be submitted to *Environmental Evidence*.

## Supplementary Information

Below is the link to the electronic supplementary material.

Supplementary Material 1. The completed ROSES (Reporting Standards for Systematic Evidence Syntheses) checklist for this systematic map protocol.

Supplementary Material 2. Test list of benchmark articles (*n* = 20) used to develop and validate the search strategy.

Supplementary Material 3. Search strategy development and final search strings.

Supplementary Material 4. Results of the iterative search syntax development and testing.

Supplementary Material 5. Data coding and extraction protocol.

## Data Availability

All data analyzed in the formulation of this protocol is accessible through the databases cited and the supplementary materials.
